# Quantitative, traceable determination of cell viability using absorbance microscopy

**DOI:** 10.1371/journal.pone.0262119

**Published:** 2022-01-19

**Authors:** Greta Babakhanova, Stephen M. Zimmerman, Laura T. Pierce, Sumona Sarkar, Nicholas J. Schaub, Carl G. Simon

**Affiliations:** 1 Biosystems and Biomaterials Division, Material Measurement Laboratory, National Institute of Standards and Technology, Gaithersburg, MD, United States of America; 2 Energy and Environment Division, Engineering Laboratory, National Institute of Standards and Technology, Gaithersburg, MD, United States of America; 3 National Center for the Advancement of Translational Sciences, National Institutes of Health, Bethesda, MD, United States of America; Mohanlal Sukhadia University, INDIA

## Abstract

Cell viability, an essential measurement for cell therapy products, lacks traceability. One of the most common cell viability tests is trypan blue dye exclusion where blue-stained cells are counted via brightfield imaging. Typically, live and dead cells are classified based on their pixel intensities which may vary arbitrarily making it difficult to compare results. Herein, a traceable absorbance microscopy method to determine the intracellular uptake of trypan blue is demonstrated. The intensity pixels of the brightfield images are converted to absorbance images which are used to calculate moles of trypan blue per cell. Trypan blue cell viability measurements, where trypan blue content in each cell is quantified, enable traceable live-dead classifications. To implement the absorbance microscopy method, we developed an open-source AbsorbanceQ application that generates quantitative absorbance images. The validation of absorbance microscopy is demonstrated using neutral density filters. Results from four different microscopes demonstrate a mean absolute deviation of 3% from the expected optical density values. When assessing trypan blue-stained Jurkat cells, the difference in intracellular uptake of trypan blue in heat-shock-killed cells using two different microscopes is 3.8%. Cells killed with formaldehyde take up ~50% less trypan blue as compared to the heat-shock-killed cells, suggesting that the killing mechanism affects trypan blue uptake. In a test mixture of approximately 50% live and 50% dead cells, 53% of cells were identified as dead (±6% standard deviation). Finally, to mimic batches of low-viability cells that may be encountered during a cell manufacturing process, viability was assessed for cells that were 1) overgrown in the cell culture incubator for five days or 2) incubated in DPBS at room temperature for five days. Instead of making live-dead classifications using arbitrary intensity values, absorbance imaging yields traceable units of moles that can be compared, which is useful for assuring quality for biomanufacturing processes.

## 1. Introduction

Cell viability is a key measurand in living cell culture-based workflows. The definition of a viable cell can include five attributes: a cell with (1) an intact membrane or the capacity for (2) metabolism, (3) motility, (4) proliferation, or (5) to react to stimuli [1]. Cell viability is one of the critical measurements that is performed throughout manufacturing to evaluate the manufacturing process itself and to ensure the quality and consistency of the product lots [2]. An accurate determination of viable cell number or percent viable cells is crucial in cell therapies and tissue-engineered constructs [3–6]. Live and dead cell counts are used to evaluate cell growth, health, and function, as well as to establish cellular therapeutic product dose [2,6–9]. It is fundamental and often under-appreciated measurement that, as noted by the cell therapy community, needs further development [10,11]. Furthermore, manufacturing numerous units of a product by different operators in different locations and with different instrumentation requires comparable measurements to assure quality. Current cell viability methods lack traceability and comparability. A survey of cell therapy stakeholders identified cell counting and viability as the measurements most in need of improvement [12].

Trypan blue (TB) dye exclusion test is one of the most common methods to assess cell viability [6,13–16]. TB can penetrate dead cells with a ruptured membrane and stain the cytoplasm blue. Viable cells with intact cell membranes are impermeable to TB and remain unstained. Current approaches for making live and dead cell classifications for TB-stained cells use the pixel intensity values in brightfield images [6,17]. However, pixel intensities vary widely between microscopes and depend on the type of microscope, optics, camera, filters, exposure time, light intensity, and other factors. Thus, if a sample is imaged on two different microscopes, the pixel intensity values in the micrographs may differ from one another in a seemingly arbitrary manner which makes the results difficult to compare.

The current work addresses the issues of metrological traceability and comparability. Metrological “traceability” is when a measurement result can be related to a reference or SI unit through a documented unbroken chain of calibrations [18]. When two measurement results are traceable to the same unit, then those measurements are considered “comparable.” This study employs absorbance microscopy since comparable absorbance measurements of a sample can be obtained when using different instruments. This is because absorbance is a ratiometric value that is determined from the fraction of the incident light that is absorbed by the sample. Further, the Beer-Lambert law enables the moles of an absorbing molecule in a solution to be determined from an absorbance measurement. The result is a value with the SI-traceable unit of moles which enables comparability.

Herein, brightfield microscopy images are utilized to generate images whose pixels are quantitative absorbance values. A user-friendly AbsorbanceQ app [19] was developed for converting brightfield images into absorbance images. The absorbance values of TB in the images can be 1) converted into moles of TB per cell or 2) used to calculate the intracellular TB molarity. In this way, absorbance imaging enables traceable and comparable live and dead cell classifications.

## 2. Materials and methods

### 2.1. Neutral density filter absorbance measurements

Neutral density (ND) filters (Edmund Optics, optical density (OD) = 0.1, 0.2, 0.3, 0.4, 0.5, 0.6, 0.7, 0.8, 0.9, 1.0, 1.3, 1.5, 2.0; blocking wavelengths (λ) 400 nm to 700 nm; Cat.# 54–460, 32–599) were used as control samples to validate absorbance microscopy calculations. To show that the method works on different microscopes, ND filters were placed on a microscope slide and images were captured using four different microscope systems: Microscope 1: Biotek Lionheart FX, Camera: GS3-U3-15S5C-C (Sony); Microscope 2: Nikon Ti2, Camera: ORCA-Flash4.0 (Hamamatsu); Microscope 3: Zeiss Axiovert S100, Camera: DCC1545M (ThorLabs, Inc.); Microscope 4: Nikon TS100, Camera: ORCA-Flash2.8 (Hamamatsu).

Brightfield images were collected using a 10x objective through a λ = 610 nm bandpass filter (Thorlabs, Cat. # 610–10, full width at half maximum = 10 ± 2 nm). The wavelength of 610 nm was selected because it is near the TB absorbance peak. For absorbance microscopy, three brightfield images were captured using the same microscope settings and shutter speed:

1. *I*_max_: blank reference sample, light shutter open (between the light source and the sample),
2. *I*_min_: blank reference sample, light shutter closed,
3. *I*: sample (ND filter placed on a glass slide), light shutter open.

An absorbance image was generated for each brightfield image using the following relationship [20]:

$$A=-{log}_{10}\left(\frac{I–I_{min}}{I_{max}–I_{min}}\right).$$ (Eq 1)

Note that the light path for *I*_max_ should include all of the components used for the experimental sample except for the actual sample itself. During absorbance imaging, when the “sample” was an ND filter supported by a glass slide, then *I*_max_ was an image of the glass slide without the ND filter. When the “sample” was cell suspension in a chamber slide, then *I*_max_ was an image of the chamber slide filled with the medium only (blank reference solution without cells and TB).

The ND filters were also measured using a spectrophotometer at λ = 598–622 nm to match the spectra of the λ = 610 nm bandpass filter used for absorbance microscopy. The OD values of ND filters were compared to those measured with the spectrophotometer, since the reported manufacturer’s value of each ND filter is the average value at λ = 350–1100 nm.

### 2.2. Trypan blue absorbance measurements

#### 2.2.1 Spectrophotometer method

Trypan blue [(3Z,3’Z)-3,3’-[(3,3’-dimethylbiphenyl-4,4’-diyl)di(1Z)hydrazin-2-yl-1-ylidene] bis(5-amino-4-oxo-3,4-dihydronaphthalene-2,7-disulfonic acid)] stock solution (Gibco, Cat.# 15250–061) was filtered using a 0.22 *μ*m sterile syringe filter (Argos Technologies, Cat.# FE32S). The concentration of TB stock solution was 4.163 mmol/L considering that the molecular weight of TB is 960.81 g/mol [21]. Thirteen TB dilutions ranging from 4.16 *μ*mol/L to 54.12 *μ*mol/L in Dulbecco’s phosphate-buffered saline, DPBS, (Gibco, Cat.# 14190–250) were prepared at room temperature (23°C) in 1 cm cuvettes (VWR, Cat.# 9700–586), sealed with a lid, and vortexed for 10 seconds. Absorbance measurements were performed using a Spark multimode reader (Tecan). DPBS was used as a blank reference solution. Three replicates of wavelength-dependent absorbance measurements were acquired using 300–900 nm light with a 1 nm interval.

#### 2.2.2 Absorbance microscopy method

Thirteen TB solutions in DPBS were prepared as described in Section 2.2.1. All absorbance images were collected in brightfield mode using a 610 nm bandpass filter (S1A Fig). Microscope optics were aligned before the experiments to avoid blur or uneven illumination. The hardware/software settings and shutter speed were picked such that intensity values were in the linear range of the camera and were not over/under-exposed (S2 Fig). The samples were placed on the microscope stage such that the light passed through the 1 cm region of the cuvettes, (S1B Fig) (BioTek Instruments, Lionheart FX). Additionally, *I*_min_ and *I*_max_ images were captured with the same settings for absorbance calculations.

### 2.3. Cell culture conditions and media

Jurkat clone E6-1 cell line (ATCC, Cat.# TIB-152) was cultured (in 37°C, 5% by volume CO_2_, humidified atmosphere) in growth medium containing 89% (by volume) RPMI-1640 Medium (1X) (HyClone, Cat.# SH30096.01), 10% (by volume) fetal bovine serum (ATCC, Cat.# 30–2020) and 1% (by volume) GlutaMAX 100X containing 200 mmol/L L-alanyl-L-glutamine dipeptide in 0.85% (mass/volume) NaCl (Gibco, Cat.# 35050–061). Cells at passages 7 to 12 were used (received at passage 5). Cells were passaged at least twice a week (every 2 to 3 days). Cell viability and counting were assessed using acridine orange and propidium iodide (AOPI) nuclear staining solution (ViaStain, Cat.# CS2-0106-5mL). A cell suspension of 10 *μ*L consisting of a 1:1 ratio of cell suspension in culture medium to AOPI staining solution was loaded into a disposable counting chamber (shown in S1C Fig, Nexcelom, Cat.# PD100) using a single channel pipette. An automated imaging cell counter (Cellometer Auto 2000) was used to image the cells, provide cell count, and assess the viability.

### 2.4. Preparing dead cells

#### 2.4.1. Fixation-killed cells

Jurkat cells were killed by fixation and then permeabilized using a fixation/permeabilization kit (CytoFix, BD Biosciences Cat.# 554715). First, the cell suspension was placed in a 15 mL tube and centrifuged at room temperature at 129× *g* (RCF) for 5 min. The supernatant was aspirated, and 1 mL of DPBS was added to wash the cells by using a gentle pipetting action. The solution was transferred to a 1.5 mL microcentrifuge tube, and the cells were spun down in a centrifuge at 244× *g* for 2 min. After the supernatant was aspirated, 1 mL of fixation buffer containing 4.2% formaldehyde (mass/volume) (Cytofix, BD Biosciences, Cat.# 51-2090KZ) was added. The solution was immediately mixed by gentle pipetting action to avoid aggregation and incubated on ice for 30 minutes. After the incubation, the cells were spun down (244× *g* for 2 min) to allow aspiration of the fixation buffer. The cells were washed with 1 mL DPBS and spun down (244× *g* for 2 min), after which the medium was aspirated. The permeabilization/wash buffer (perm/wash, BD Biosciences, Cat.# 51-2091KZ) supplied as a 10X stock solution that contained FBS and saponin was then diluted to 1X perm/wash buffer in DPBS (1:10). Saponin was necessary to permeabilize the cell membrane for TB staining. The cells were re-suspended in 1 mL of 1X perm/wash buffer in a 1.5 mL microcentrifuge tube and placed on a rocker for 15 min at room temperature. Afterward, the solution was spun down (244× *g* for 2 min) and the supernatant was aspirated. The cells were washed with 1 mL DPBS by gentle pipetting action and spun down (244× *g* for 2 min). Finally, the supernatant was aspirated, and the cells were re-suspended in 1 mL of cell culture medium. This procedure yields dead cells that are stable for several hours and do not aggregate.

#### 2.4.2. Heat-shock killed cells

A digital dry bath (Isotemp, Fisher Scientific) was pre-warmed to 70°C. Cell death was induced by placing the Jurkat cell suspension (in cell culture medium) in a conical 1.5 mL microcentrifuge tube and heating it at 70°C for 10 minutes. The cells were then spun down (244× *g* for 2 min) to allow aspiration of the spent media. The cells were immediately mixed with 1 mL DPBS by gentle pipetting action to avoid aggregation. The heat-shock killed cells were kept at room temperature and were stable for the duration of the experiments (several hours).

#### 2.4.3. Overgrown cells and cells incubated in DPBS

To mimic batches of low viability cells that may be encountered during a cell manufacturing process, cells were 1) overgrown for five days in the cell culture incubator (‘Overgrown-5d’) or 2) incubated in DPBS for five days outside of the cell culture incubator (‘DPBS-RT-5d’). ‘Overgrown-5d’ Jurkat cells at passage 9 were produced by culturing live-cell suspension in the cell culture incubator (in 37°C, 5% by volume CO_2_, humidified atmosphere) for five days in a T75 flask with no media change or replenishment. The cell density on day five was 5.21x10^6^ cells/mL. ‘DPBS-RT-5d’ Jurkat cells at passage 9 were produced by first placing the live-cell suspension in a 15 mL tube and centrifuging it at room temperature at 129× g for 5 min. The supernatant was aspirated and the cells were resuspended in DPBS and incubated at room temperature (22°C, outside of the cell culture incubator) for five days.

#### 2.4.4. Assumptions

The assumptions are that live-cell treatments have mostly live cells with a few dead cells, while fixation- and heat shock-killed cell treatments have 100% dead cells. Further, it is assumed that the ‘Overgrown-5d’ and ‘DPBS-RT-5d’ treatments contain an unknown number of live and dead cells, but should have fewer live cells and more dead cells than the live-cell treatment. These assumptions are important for evaluating the results presented herein.

### 2.5. Absorbance microscopy of Jurkat cells

Seven different treatments of Jurkat cells were prepared for absorbance microscopy experiments.

1. ‘Live cells in DPBS’, (‘LD’): Live cell suspension was placed in a 15 mL tube and centrifuged at room temperature at 129× *g* for 5 min. The supernatant was aspirated, and 1 mL of DPBS at room temperature was added and mixed by gentle pipetting action. This sample does not contain any TB.
2. ‘Dead cells in DPBS’, (‘DD’): the resultant solution according to the protocol described in Section 2.4.1 or Section 2.4.2. This sample does not contain any TB.
3. ‘Live cells in TB’, (‘LT’): Live cell suspension was placed in a 15 mL tube and centrifuged at room temperature at 129× *g* for 5 min. The supernatant was aspirated, and 1 mL of DPBS at room temperature was added and mixed by gentle pipetting action. Immediately before imaging, filtered TB solution was added to live cells in DPBS at a 1:4 ratio and mixed by gentle pipetting action (0.833 mmol/L final TB concentration during staining).
4. ‘Dead cells in TB’, (‘DT’): Immediately before imaging, filtered TB solution was added to the DPBS solution with dead cells (prepared according to the protocols described in Section 2.4.1 or Section 2.4.2) at 1:4 ratio.
5. ‘Live and dead cells in TB’: Live and dead (either fixation-killed or heat shock-killed) cells in DPBS were prepared and stained with AOPI staining solution in two separate 1.5 mL microcentrifuge tubes. Live/dead cell counting was performed using an automated Nexcelom cell counter, after which the cell density of live/dead cells in DPBS was adjusted so that live and dead cells in DPBS had identical concentrations. Next, an equal amount of live and dead cells in DPBS was transferred into a new 1.5 mL microcentrifuge tube. Immediately before imaging, filtered TB solution was mixed with live and dead (1:1 ratio) cells in DPBS at a 1:4 ratio and mixed by gentle pipetting action (to avoid shear forces that may damage the cells). A homogeneous mixture of live and dead cells is crucial for the accurate determination of live/dead cell count.
  For the heat-shock killing method, the five treatments were performed on two different microscopes to assess the comparability. Each experiment was repeated three times for a total of nine trials: three heat-shock on microscope 1, three heat-shock on microscope 2, and three fixation experiments on microscope 1. Approximately 10 *μ*L of each sample was pipetted into a glass chamber slide (NC-Slide, A8, Chemometec) (S1C Fig). For absorbance microscopy, glass slides work better than plastic slides, since the latter may be uneven and deformed which may cause defocusing in some regions in the field of view during imaging. For each experiment, four *I*_max_ and four *I*_min_ images of four different fields of view were captured, then *I* images of ten different fields of view were captured. Cells rapidly sediment during handling and it is crucial to adequately mix cell suspensions with gentle pipetting action each time an aliquot of cell suspension is to be dispensed. Since TB may be toxic [15], it was mixed with cells immediately before imaging and the absorbance imaging process was performed as quickly as possible (3–5 min).
6. ‘Overgrown-5d’ cells: The overgrown Jurkat cells were placed in a 1.5 mL microcentrifuge tube. The cells were then spun down (244× *g* for 2 min) to allow aspiration of the spent media. The cells were immediately mixed with 1 mL DPBS by gentle pipetting action to avoid aggregation. Immediately before imaging, the filtered TB solution was mixed with the cells in DPBS at a 1:4 ratio and mixed by gentle pipetting action.
7. ‘DPBS-RT-5d’ cells: Immediately before imaging, the filtered TB solution was mixed with the ‘DPBS-RT-5d’ cells in DPBS at a 1:4 ratio and mixed by gentle pipetting action.

### 2.6. Image analysis

**Fig 1:**
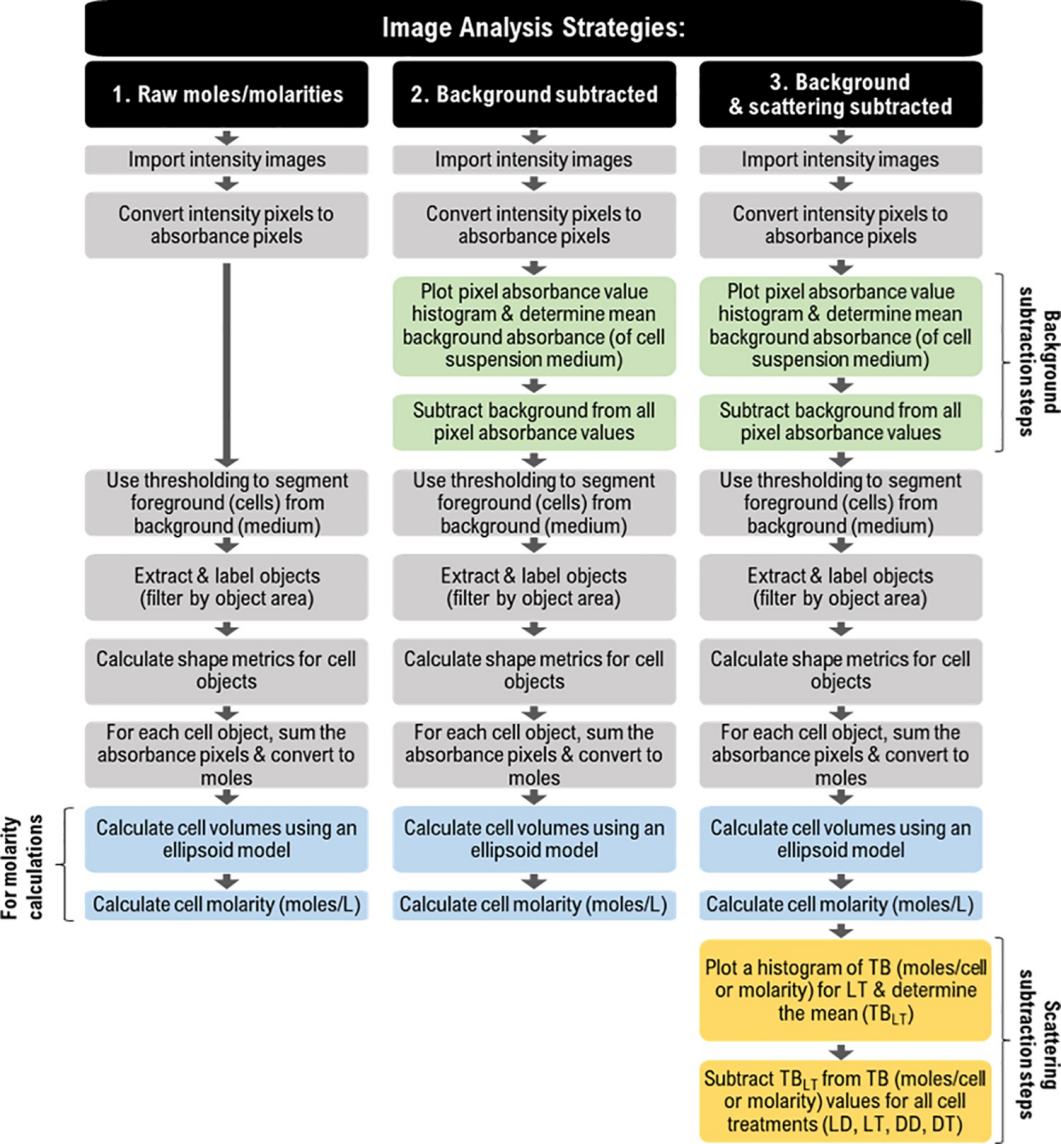
Flow chart outlining three strategies of the image analysis workflow. As the complexity of the image analysis workflow increases, so does the accuracy of the intracellular content of trypan blue (TB) calculation. Abbreviations: LD = live cells in Dulbecco’s phosphate-buffered saline (DPBS) solution, LT = live cells in TB solution mixed with DPBS at a 1:4 (TB:DPBS) ratio, DD = dead cells in DPBS, DT = dead cells in TB solution mixed with DPBS at a 1:4 ratio (TB:DPBS) and TB_LT_ = TB content in LT sample.

Image analysis in MATLAB software was performed for three different strategies of calculating the TB concentration in cells (Fig 1). The goal is to segment the cells and then use the absorbance values to determine moles of TB within each cell. The image analysis complexity increases with each strategy to increase accuracy. Lastly, a control sample consisting of a live and dead mixture of cells (1:1) in TB is assessed as a test of the approach. Between 637 and 3616 total cells were counted for each sample in the nine experiments that were conducted.

#### 2.6.1. Strategy #1: “Raw moles/molarities”

This is the image processing algorithm that calculates intracellular TB content but does not account for background or scattering contributions.

Step #1: Import *I*_max_, *I*_min_, *I* 16-bit.tif images into MATLAB. For better accuracy, we imported four different *I*_max_ and four different *I*_min_ images and used the average images for the rest of the algorithm. For each experiment, ten *I* images corresponding to different fields of view in a sample were analyzed.
Step #2: Convert intensity pixels into absorbance pixels using Eq 1. An open-source application called AbsorbanceQ has been deployed to perform this conversion [19].
Step #3: Convert the matrix of absorbance values to a grayscale absorbance image.
Step #4: Compute a locally adaptive threshold with a sensitivity factor specified for each image [22].
Step #5: Create a binary image from the grayscale image by replacing all values above the determined threshold with ones and setting all other values to zeros.
Step #6: Extract all connected components (objects) from a binary image where the area is in the specified range. In this work, the minimum and maximum range were set to the pixel values which correspond to the circular area of Jurkat cells having a radius between 4 *μ*m and 16 *μ*m. Trace and label connected components in a binary image (connectivity = 8).
Step #7: Measure properties of connected object regions: area, absorbance values, minimum Feret diameter, and maximum Feret diameter.
Step #8: For each extracted object sum all the absorbance pixels and convert them to moles. A detailed example of this calculation is shown in S3 Fig. The analysis is complete if the desired unit is mol/cell. Execute steps #9 and #10 if the desired unit is molarity (moles/L).
Step #9: Calculate the object (cell) volumes using the volume of an ellipsoid equation: Volume = (4/3)(*πabc*), where *a*, *b* and *c* are the semiaxis lengths for the three major axes. One-half of the maximum Feret diameter was assigned to *a*, while one-half of the minimum Feret diameter was assigned to *b* and *c*. Knowledge of the pixel dimensions is required to determine the volume in liters.
Step #10: Calculate cell molarity (moles/L).
Step #11: Save the resultant output data (e.g. mol/cell, cell volumes, cell molarities).

Omit steps #9 and #10 if using mol/cell (include if molarity is needed).

#### 2.6.2. Strategy #2: “Background subtracted”

This is the image processing algorithm that calculates intracellular TB content and includes a background subtraction (but not a scattering subtraction).

Step #1: Execute Steps #1,#2 outlined in Strategy #1.
Step #2: Plot a histogram of the pixel absorbance values. In our case, the data exhibits bimodal distribution corresponding to the pixels that fall within a cell (higher absorbance) and pixels corresponding to the surrounding medium/background (lower absorbance). Fit a Gaussian mixture (bimodal) model to determine the mean absorbance of the background.
Step #3. Subtract the mean value corresponding to the background peak of the histogram (from step #2) from the absorbance image. This will cause both peaks to shift and the background peak should center over zero absorbance.
Step #4: Execute Steps #3–11 outlined in Strategy #1.

#### 2.6.3. Strategy #3: “Background & scattering subtracted”

This is the image processing algorithm that calculates the intracellular TB content and includes background and scattering subtraction.

Step #1: Execute all steps outlined in Strategy #2.
Step #2: Plot a histogram of calculated TB “mol/cell” or “molarity” for LT using the Strategy #2 output and fit a histogram (Gaussian) to determine the mean (*μ*_*LT*_*noBG*_).
Step #3: Subtract *μ*_*LT*_*noBG*_ from the output data of Strategy #2 (TB mol/cell or molarity) for LD, LT, DD, and DT samples. (Note that subtracting *μ*_*LT*_*noBG*_ eliminates the need for the user to perform control experiments using cells in DPBS).

#### 2.6.4. Live and dead cell counting for “Live and dead cells in TB”

Step #1: Plot and fit the combined LT and DT data to determine the minimum, *C*_min_, that separates live and dead cells. Here, the Lorentzian fit was used for mol/cell and the Gaussian fit for molarity histograms.
Step #2: Execute Strategy #3 for the ‘Live and dead cells in TB’ dataset.
Step #3: Count the number of cells below and above *C*_min_ threshold to identify the number of live and dead cells, respectively.

#### 2.6.5. Live and dead cell counting for ‘Overgrown-5d’ and ‘DPBS-RT-5d’

Step #1: Execute steps outlined in Section 2.6.3. for LT, ‘Overgrown-5d’ and ‘DPBS-RT-5d’ samples.
Step #2: For LT, fit the live cell peak using Gaussian fit and determine the upper value for three standard deviations (3*σ*) of the mean *μ*_*LT*_*noBG*_. This value will be used as the cutoff between live and dead cells and includes 99.9% of the cells in the live cell Gaussian fit.
Step #3. Count the number of cells below and above the 3*σ* threshold to identify the number of live and dead cells, respectively in ‘Overgrown-5d’ and ‘DPBS-RT-5d’ samples.

## 3. Results

### 3.1. Absorbance microscopy validation

**Fig 2:**
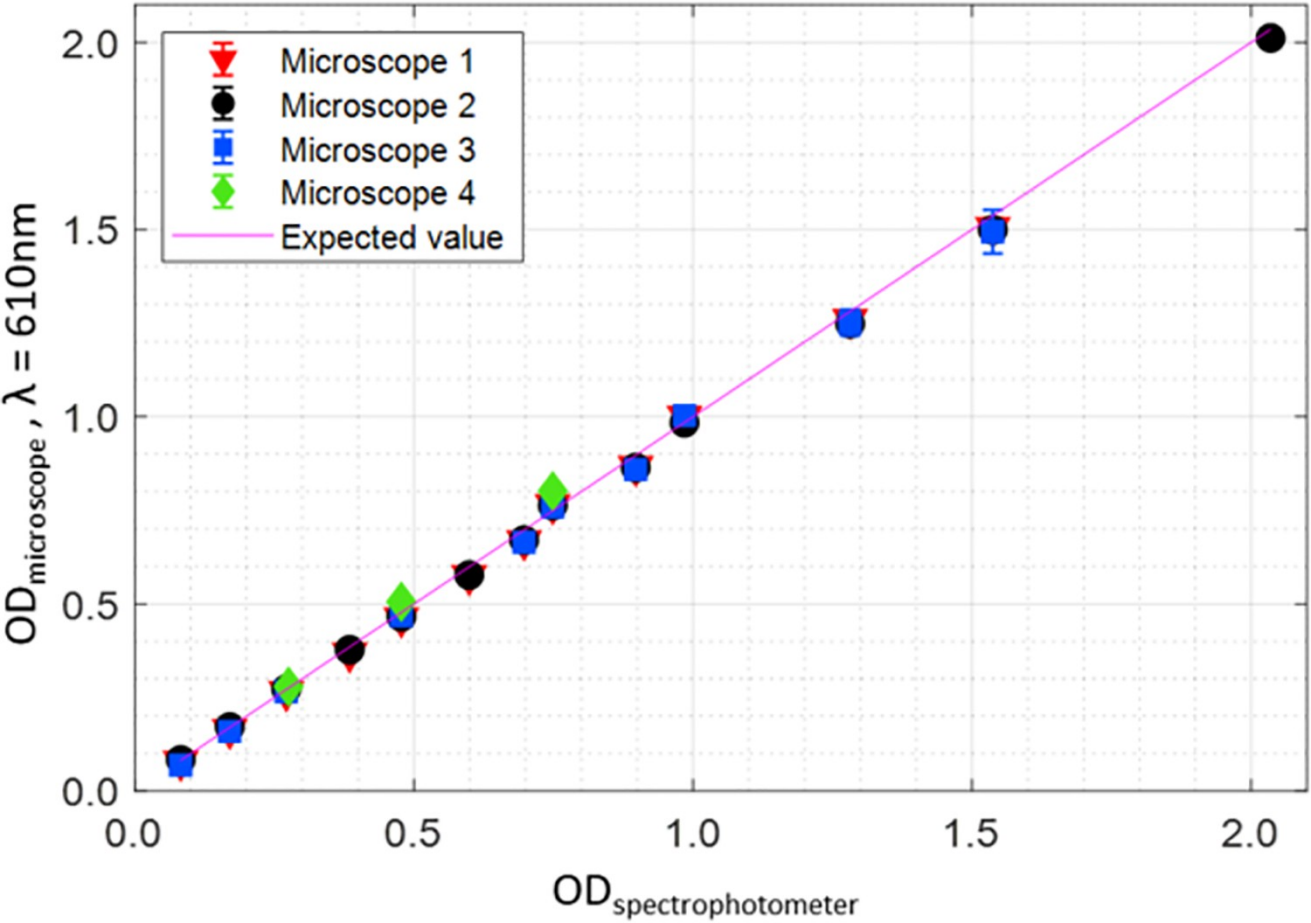
Validation of absorbance microscopy method. Absorbance microscopy data of ND filters plotted against the measured OD values using a spectrophotometer (average OD at λ = 598 to 622 nm). There is a strong correlation between OD measured by each microscope and the spectrophotometer indicating that the brightfield microscopy can be used to achieve comparable absorbance measurements even when microscopy instruments differ. Imaging was performed using four different microscopes equipped with different cameras and a 610 nm bandpass filter. The error bars represent the standard deviation.

For the four microscopes, the mean absolute percentage deviation between experimental results and the ND filter OD values is 3% (Microscope #1), 2% (Microscope #2), 4% (Microscope #3), 5% (Microscope #4) (Fig 2). These results demonstrate that a brightfield microscope can be used to make accurate absorbance measurements. Each sensor in the camera array serves as a tiny spectrophotometer to yield an absorbance image composed of numerous quantitative measurements.

**Fig 3:**
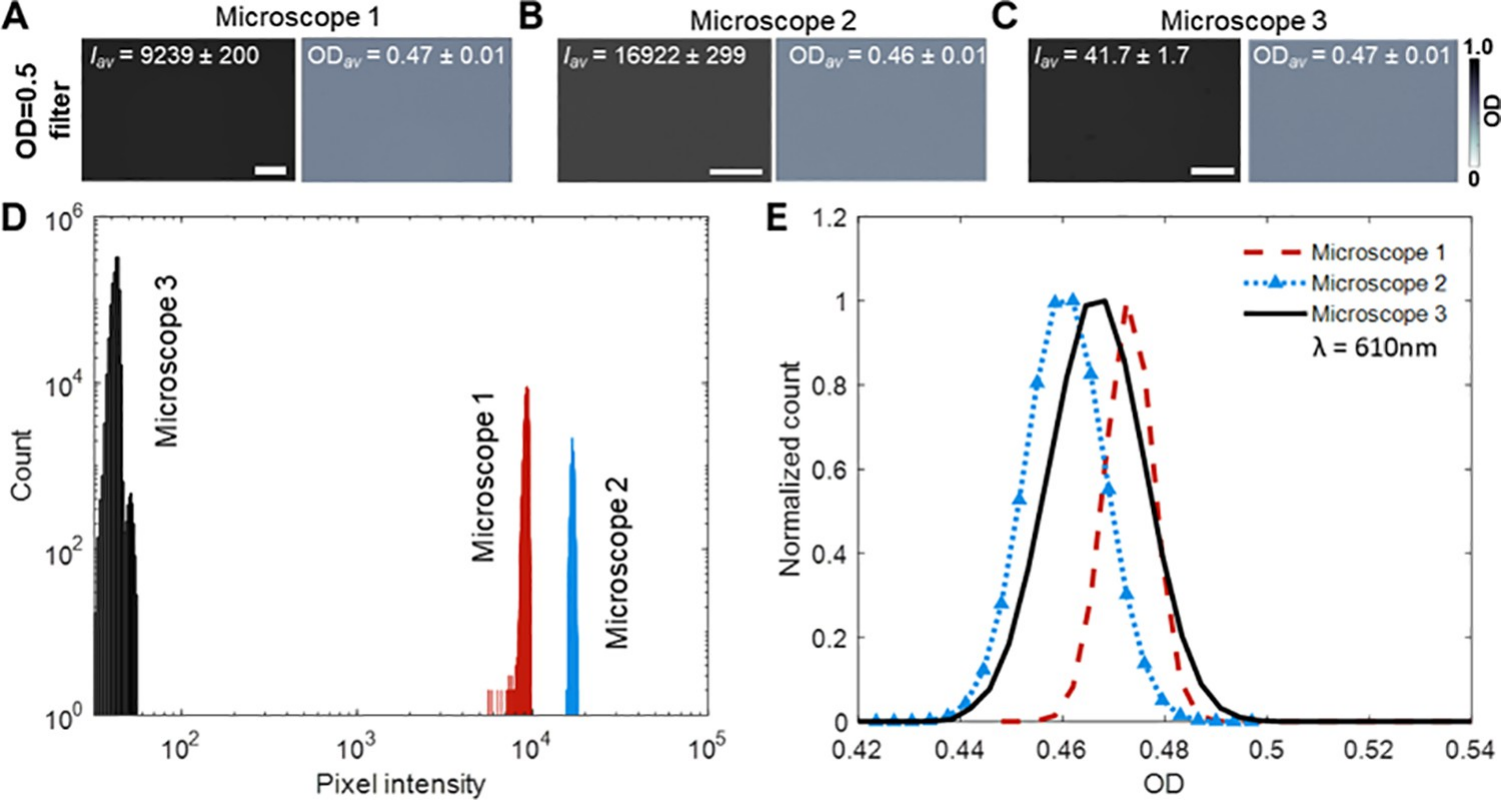
Demonstrating comparability of the absorbance imaging. **(A-C)** Using three different microscopes, a brightfield image of an OD = 0.5 neutral density (ND) filter was captured as shown on the left of each panel. Corresponding absorbance images are shown on the right. The average pixel values of intensity and OD are indicated on each image. Microscope 1: Biotek Lionheart FX, Microscope 2: Nikon Ti2; Microscope 3: Zeiss Axiovert S100; Microscopes 1 and 2 yield 16-bit while Microscope 3 yields 8-bit images. **(D)** Histograms of pixel intensities of the brightfield images do not overlap when imaging the same ND filter, which shows that pixel intensities cannot be used to compare data when using different microscopes. **(E)** Overlapping histograms of the OD values show that the absorbance microscopy method can be used to compare data collected on different microscopes (the number of pixels in each image presented on the *y*-axis count was normalized since the image dimensions for each microscope differ). All microscopes were equipped with a 610 nm bandpass filter. Scale bars: 200 *μ*m.

A demonstration of how absorbance imaging can improve data comparability is presented in Fig 3. First, brightfield images were captured of an OD = 0.5 ND filter and the histograms of pixel intensities were plotted (Fig 3D). The histograms illustrate how the intensity values differ between the three microscope systems. Next, the brightfield images were converted to absorbance images, and absorbance values were plotted as histograms to reveal better agreement between the microscopes (Fig 3E). Pixel intensities from brightfield images captured in different microscopes vary in a seemingly arbitrarily manner due to variations in microscope components and settings. In contrast, the division step in Eq 1 “normalizes” pixel intensities so that comparable absorbance data can be collected on different microscopes [20].

### 3.2 Using a microscope to measure trypan blue absorbance

**Fig 4:**
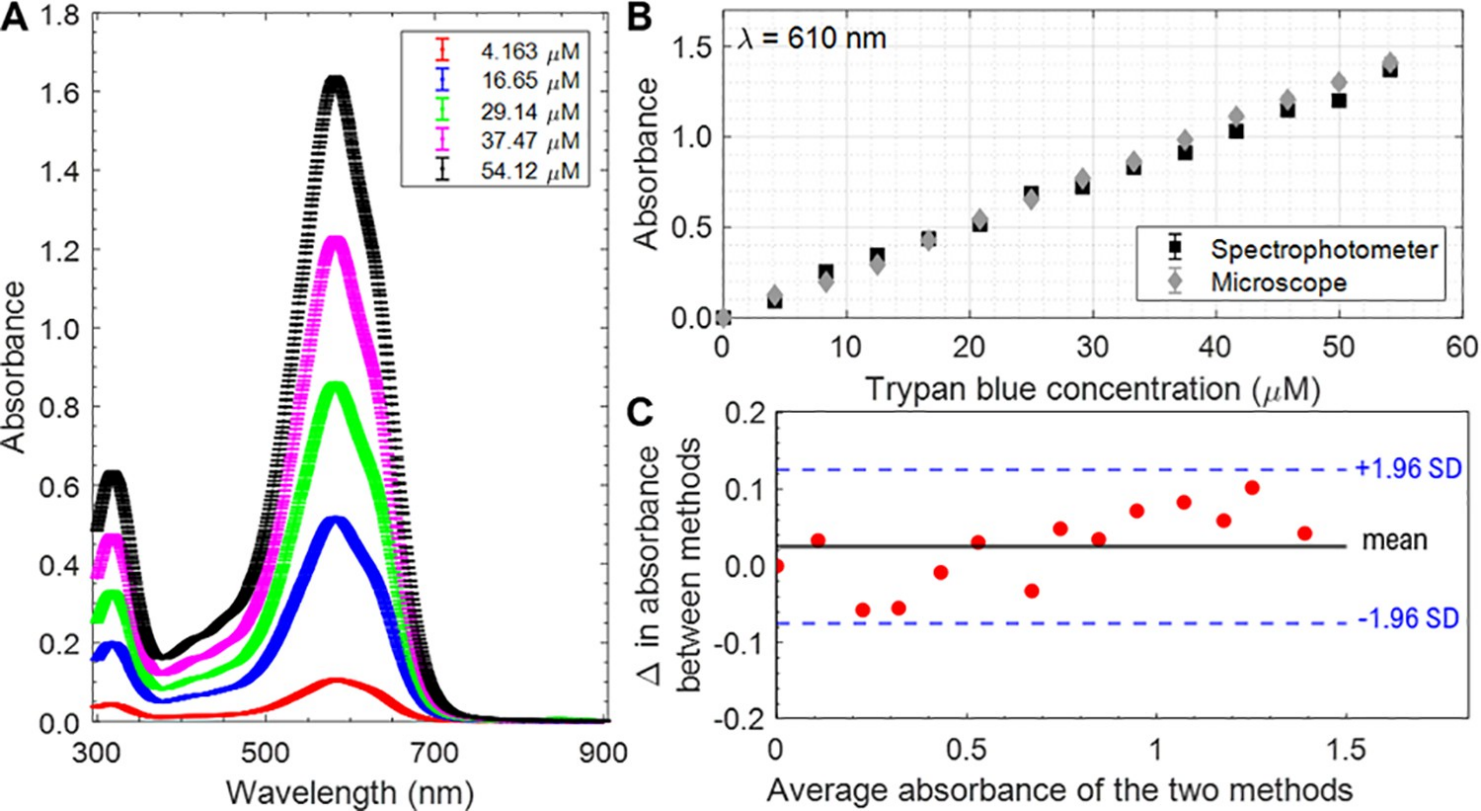
Trypan blue (TB) absorbance measurements. **(A)** TB absorbance spectrum in Dulbecco’s phosphate-buffered saline (DPBS) measured using a spectrophotometer. Each data point is an average of three replicates (3 readings of the same cuvette) and error bars are standard deviation (SD). **(B)** TB absorbance at *λ* = 610 nm measured using a spectrophotometer (black squares) and absorbance microscopy (gray diamonds). The error bars represent SD (3 readings of the same cuvette). **(C)** Bland–Altman plot for assessing agreement between two methods presented in panel (B), where the solid black line denotes the bias between the mean differences (Δ) and dashed blue lines indicate ± 1.96 SD, which cover 95% of the values.

The TB absorbance spectrum at a series of concentrations was measured in a spectrophotometer and shows increasing absorbance with increasing TB concentration (Fig 4A and 4B). Measurements of the absorbance of TB dilutions with both the spectrophotometer and the microscope (in brightfield mode) are plotted in Fig 4B. The λ = 610 nm bandpass filter for absorbance microscopy experiments was selected since 610 nm is near the TB absorption peak. Both plots in Fig 4B show a linear response and the results are in close agreement. The agreement between the two methods is demonstrated by the Bland-Altman plot in Fig 4C, where the differences in absorbance are plotted against the averages [23].

To determine the concentration of TB in cells using absorbance microscopy, the molar absorption coefficient (*ε*) at a specific wavelength must be determined. It was obtained using the Beer-Lambert Law which establishes the relationship between absorbance and chemical concentration:

A(λ)=ε(λ)cl, (Eq 2)

where *c* is the concentration and *l* is the path length of the beam of light. The concentration (*c*) of the TB solutions was known, the pathlength (*l*) was 1 cm, and the absorbance at 610 nm (*A*) was measured. Molar absorption coefficient (*ε*_610*nm*_) was calculated 1) using the spectrophotometer data and 2) using the absorbance microscopy data: *ε*_spectrophotometer_ = 2555 ± 212 m^2^/mol and *ε*_microscope_ = 2609 ± 157 m^2^/mol (mean ± standard deviation, SD); difference of 2.1%

### 3.3. Determination of intracellular TB concentration

**Fig 5:**
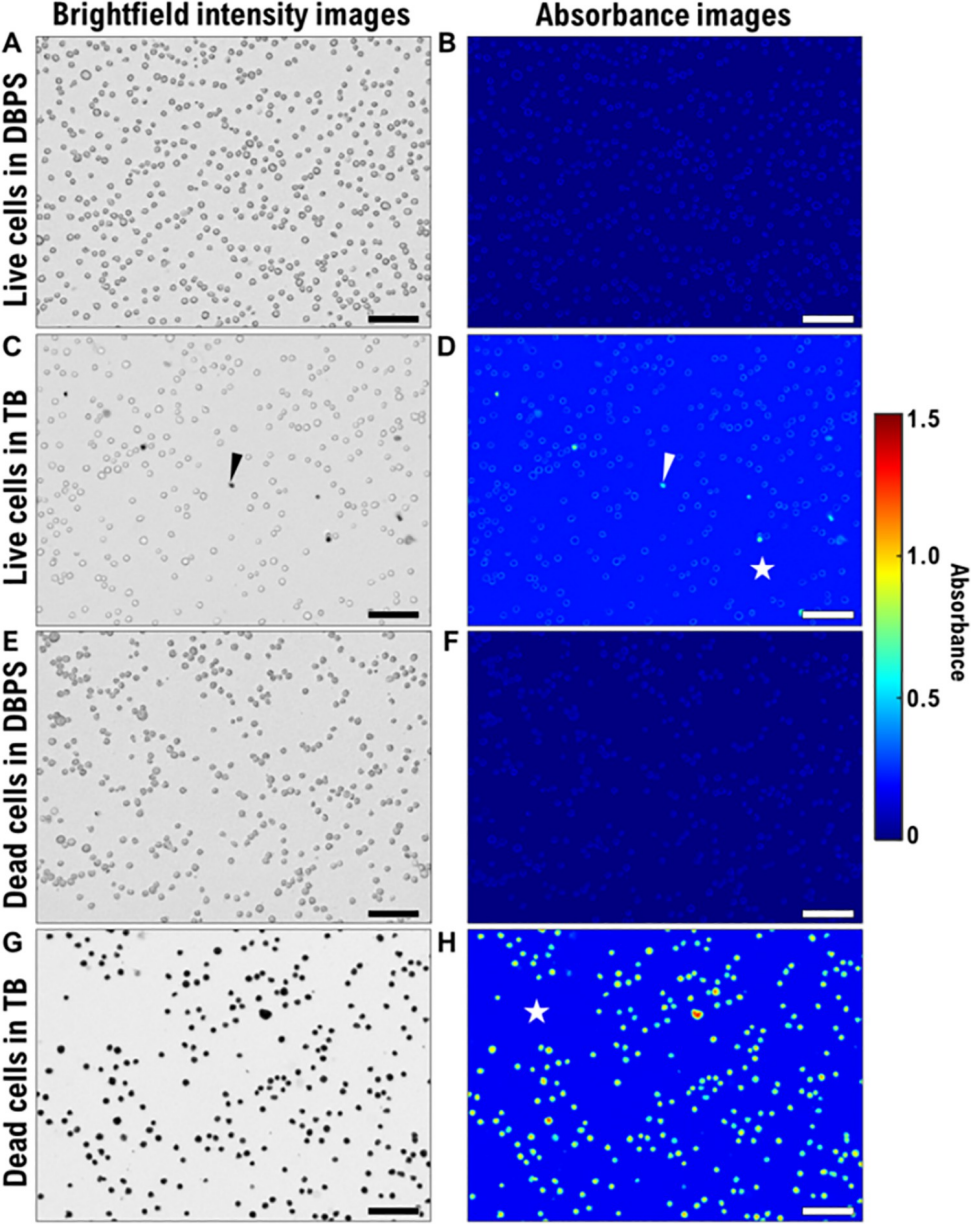
Brightfield intensity images and corresponding absorbance images of four Jurkat cell treatments. **(A,B)** LD: Live cells in Dulbecco’s phosphate-buffered saline (DPBS) solution; **(C,D)** LT: Live cells in trypan blue (TB) solution mixed with DPBS at a 1:4 (TB:DPBS) ratio; **(E,F)** DD: Dead cells in DPBS; **(G,H)** DT: Dead cells in TB solution mixed with DPBS at a 1:4 ratio (TB:DPBS). Dead cells were produced using the heat-shock cell killing method. Arrowheads in panels C and D indicate a dead cell in the LT sample. The stars in panels D and H indicate the medium which contains TB solution mixed with DPBS at a 1:4 ratio (TB:DPBS) and absorbs light giving it a lighter shade of blue (higher absorbance value as compared to panels B and F). Scale bars: 100 *μ*m.

Fig 5 shows the brightfield intensity images and the corresponding absorbance images of four Jurkat cell treatments using the heat-shock killing method. The corresponding intensity and absorbance images for the fixation killing experiment are shown in S4 Fig. The DPBS samples were prepared as controls to assess the contribution of scattering. In the DPBS images (Fig 5A, 5B, 5E and 5F; S4A, S4B, S4E and S4F Fig), cell edges are visible in both the live and dead cell preparations. The non-zero absorbance contribution may be attributed to cell scattering. In the LT (Fig 5C and 5D; S4C and S4D Fig), the background medium (indicated as a star in Fig 5D and S4D Fig) contains TB and thus has higher absorbance. The LT images have a few dead cells stained with TB (see arrowheads in Fig 5C and 5D; S4C and S4D Fig), since healthy cell cultures usually contain some dead cells. Cells in DT are all dead (Fig 5G and 5H; S4G and S4H Fig) and take up TB, appear dark in the brightfield images, and have high absorbance values in the absorbance images.

**Fig 6:**
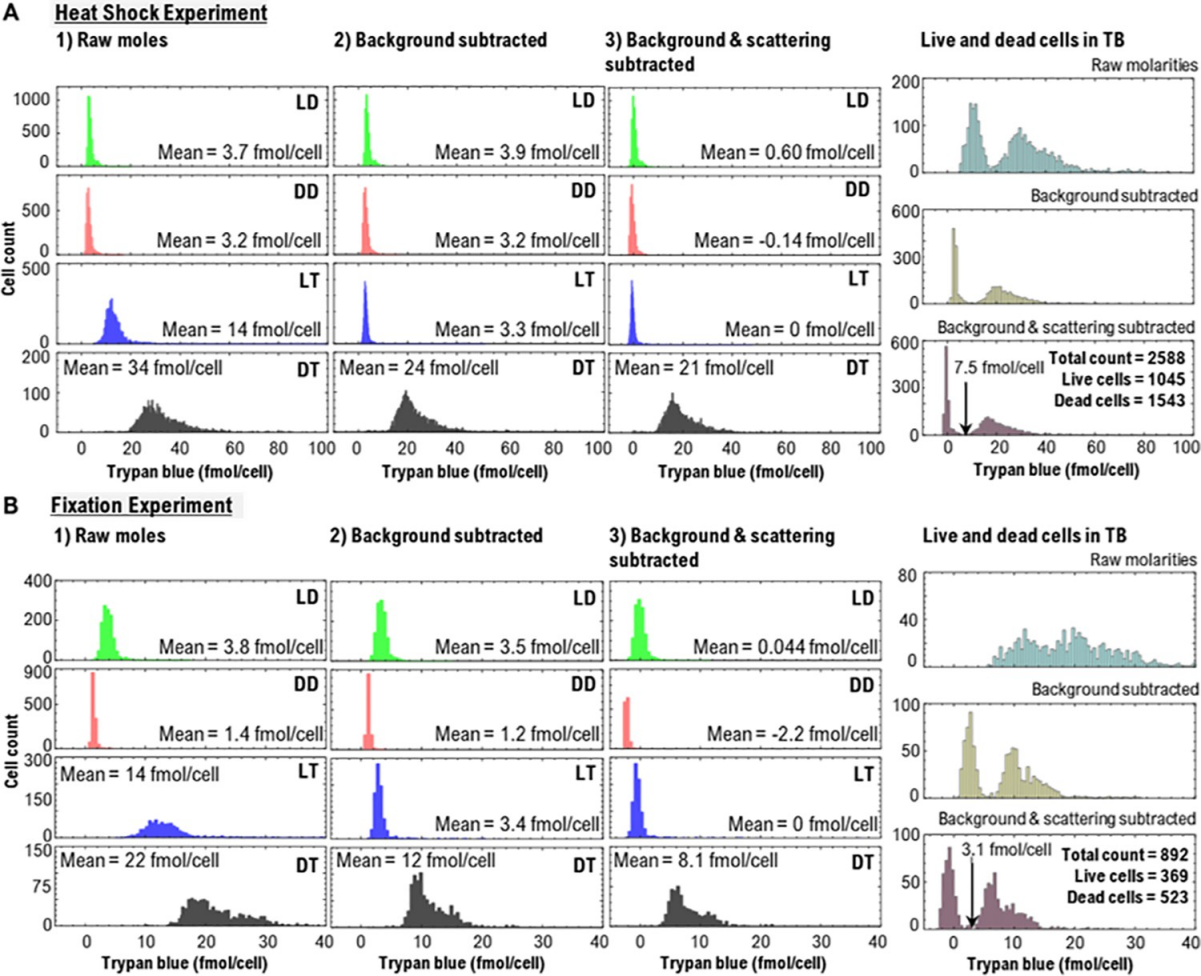
Determination of intracellular trypan blue (TB) content (mol/cell) using absorbance images. Absorbance microscopy output data for **(A)** heat-shock and **(B)** fixation experiments illustrating the histograms of the intracellular moles of TB using three different image processing strategies: 1) ‘Raw moles,’ 2) ‘Background subtracted,’ and 3) ‘Background & scattering subtracted.’ The far-right column reveals the live and dead cell counts for the control ‘Live and dead cells in TB’ experiment, where an equal number of live and dead cells were mixed in TB+DPBS solution. The arrow in the far-right column denotes the calculated threshold using LT and DT samples using image processing strategy #3. The threshold is used to calculate live cells (cells below the threshold) and dead cells (cells above the threshold). Abbreviations: LD = live cells in Dulbecco’s phosphate-buffered saline (DPBS) solution, LT = live cells in trypan blue (TB) solution mixed with DPBS at a 1:4 (TB:DPBS) ratio, DD = dead cells in DPBS, and DT = dead cells in TB solution mixed with DPBS at a 1:4 ratio (TB:DPBS). The histograms in panels **(A)** and **(B)** show that the intracellular uptake of TB is different depending on the cell-killing method.

The TB content (mol/cell) for five different treatments (LD, DD, LT, DT, and ‘Live and dead cells in TB’) and two different cell killing methods is shown in Fig 6. The intracellular TB concentrations (mmol/L) were also calculated for the same treatments and are presented in S5 Fig. The histograms of the results are presented for three different image processing strategies outlined in Section 2.6. The image analysis complexity increases to increase accuracy.

### 3.4. “Raw moles” image processing strategy

The “Raw moles” of LD and DD samples have non-zero TB content (ranging from ~1.4 fmol/cell to 3.8 fmol/cell), even though these samples have not been stained with TB (Fig 6). This is due to light scattering at the cell edges. The LT samples have TB values of ~14 fmol/cell, even though these cells have not been killed. This is due to both light scattering and the TB in the medium. TB uptake in DT samples is the highest (ranging from ~22 fmol/cell to 34 fmol/cell) due to light scattering, TB in the medium, and the TB inside the dead cells.

### 3.5. “Background subtracted” image processing strategy

Here the aim was to correct the background absorbance. Background absorbance is defined as the absorbance in an image in the regions that fall outside of the cell boundaries where only the suspension medium (DPBS+TB) lies within the path length of the light (star in Fig 5D and 5H, S4D and S4H Fig). For LD and DD samples the suspension medium is DPBS which has little absorbance, thus the background correction has little effect on the intracellular TB content. In contrast, the background subtraction has a large effect on LT and DT, both of which have TB in the medium. In Fig 6A for heat-shock-killed cells, LT_Raw molarities_ is 14 fmol/cell while LT_Background subtracted_ drops to 3.3 mmol/cell. Thus, the background subtraction helps improve the accuracy of the intracellular TB molarity measurement.

### 3.6. “Background & scattering subtracted” image processing strategy

We assume that subtracting the absorbance values due to light scattering in LT samples from each cell will predominantly reveal its absorbance due to the intracellular uptake of TB. In Fig 6A, LT_Background subtracted_ is 3.3 fmol/cell, which when subtracted from itself yields LT_Background & scattering subtracted_ of 0 fmol/mol. Likewise, DT drops from 24 fmol/cell in “Background subtracted” to 21 fmol/cell in “Background & scattering subtracted.” The “Background & scattering subtracted” strategy yields intracellular TB content close to 0 fmol/cell for unstained dead cells (DD) and live cells (LD, LT), which is sensible since these cells should not contain TB.

### 3.7. Results of nine experiments to determine intracellular TB content

**Fig 7:**
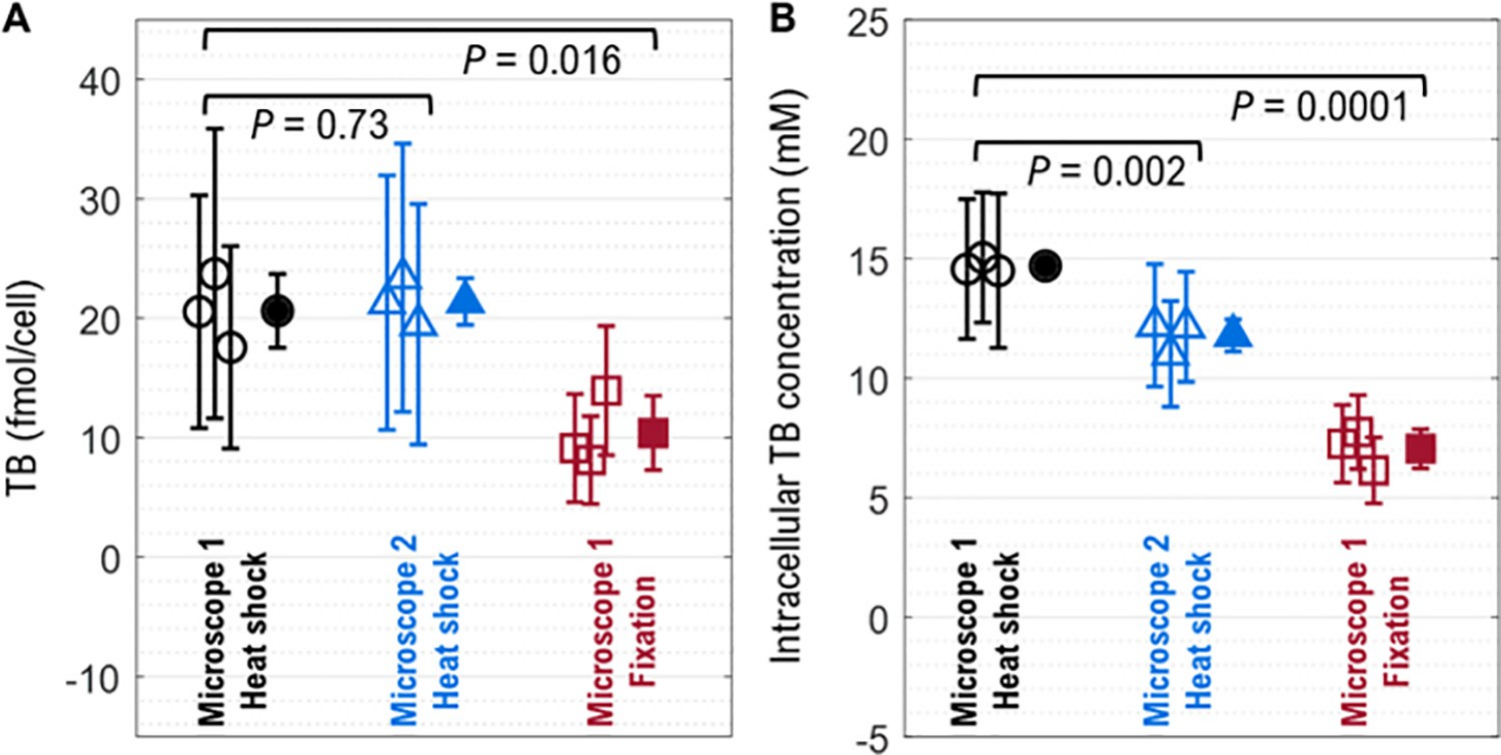
Trypan blue (TB) measurements in dead Jurkat cells after implementing the “background and scattering subtracted” image processing routine for three different datasets. Results are presented in **(A)** mol/cell and **(B)** mmol/L. Open symbols indicate triplicate experiments (same sample preparation, data acquired on three different days) of heat-shock treatment using Microscope 1 (black circles), heat-shock treatment using Microscope 2 (blue triangles), and fixation treatment using Microscope 1 (red squares). Microscope 1: Biotek Lionheart FX, Microscope 2: Nikon Ti2. The filled symbols represent the mean of three replicates. The error bars represent the standard deviation. The heat-shock killed dead cells uptake twice the TB as compared to the fixed dead cells. *P*-values from t-tests are shown below brackets.

The intracellular TB content of DT samples expressed in mol/cells for the nine experiments is shown in Fig 7A. The coefficients of variation (CV [SD/mean]) between triplicate results for each data set are 15% (Heat-Shock, Microscope 1), 9% (Heat-Shock, Microscope 2), and 30% (Fixation, Microscope 1). For the heat-shock killed cells, the mean intracellular content of TB per cell for three replicates differed between microscope 1 (20.6 fmol/cell) and microscope 2 (21.4 fmol/cell) by 3.8%. As measured on microscope 1, the difference between TB content for heat-shock-killed cells (20.6 fmol/cell) and fixation-killed cells (10.4 fmol/cell) was 49.6% (two-tailed unpaired t-test: *P* = 0.016). The intracellular TB molarities are presented in Fig 7B. For the heat-shock killed cells, TB molarity differed between microscope 1 (14.7 mmol/L) and microscope 2 (11.8 mmol/L) by 21.9%.

### 3.8. Mixtures of live and dead cells

**Fig 8:**
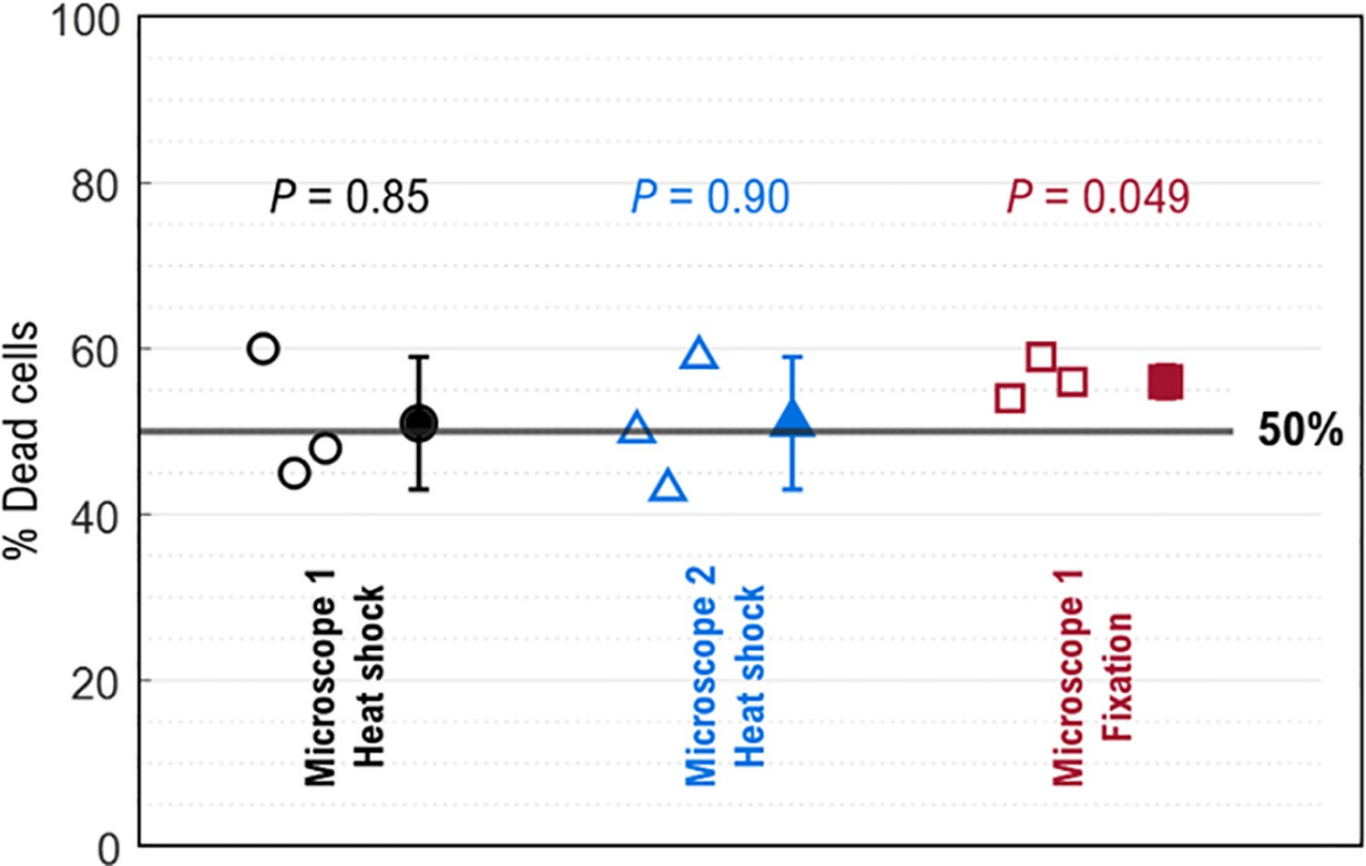
Percent dead cells of three different experimental conditions of ‘Live and dead cells in TB’ experiment. Open symbols indicate triplicate experiments (same sample preparation, data acquired on three different days) of heat-shock treatment using Microscope 1 (black circles), heat-shock treatment using Microscope 2 (blue triangles), and fixation treatment using Microscope 1 (red squares). Microscope 1: Biotek Lionheart FX, Microscope 2: Nikon Ti2. The filled symbols represent the mean of three replicates and the corresponding error bars indicate standard deviation. Above the data are *P*-values from a one-sample t-test (two-tailed) where a low *P*-value indicates an increased probability that the sample mean is different from the 50% hypothesized value.

Absorbance microscopy was used to count live and dead cells in a live-dead cell mixture. To establish a thresholding process that could be used on an “unknown,” the LT and DT histograms for “Background & scattering subtracted” (Fig 6) were combined, and the minimum, *C*_min_, between the live and dead was determined (see Sec. 2.6.4). This value was 7.5 fmol/cell for a heat-shock experiment in Fig 6A and 3.1 fmol/cell for the fixation experiment in Fig 6B as indicated in the far-right panels. Using this approach, results from the nine experiments are shown in Fig 8 where a theoretically correct result is 50% dead cells. Results range from 43% to 60% and the mean was 53% (SD = 6.4%, n = 9). A dead cell count of slightly higher than 50% might be expected, since measuring stained dead cells is more reliable than analyzing live cells which do not stain and have a lower signal-to-noise ratio. Additionally, suspension in DPBS at ambient conditions may cause cells to die during the sample preparation process.

### 3.9. Mimicking batches of low-viability cells that may be encountered during a cell manufacturing process

**Fig 9:**
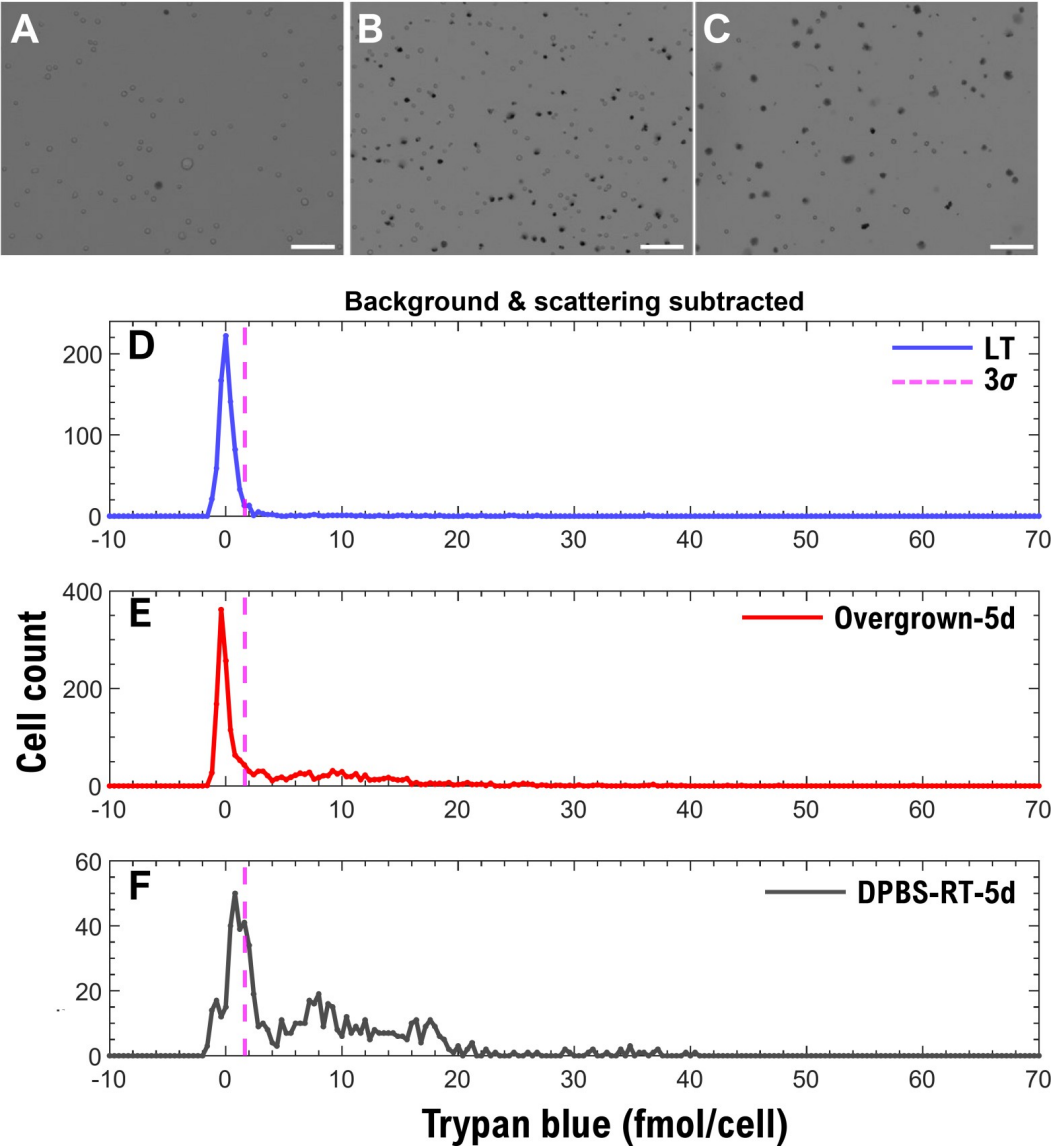
Intracellular trypan blue (TB) content (mol/cell) for cells overgrown for five days and cells incubated in DPBS at room temperature for five days. Brightfield images of **(A)** LT, **(B)** ‘Overgrown-5d’ and **(C)** ‘DPBS-RT-5d’ samples. Absorbance microscopy histograms for **(D)** LT, **(E)** ‘Overgrown-5d’ and **(F)** ‘DPBS-RT-5d’ samples indicate the TB uptake by Jurkat cells (the background TB and scattering are subtracted). Abbreviations: LT = live cells in trypan blue (TB) solution mixed with Dulbecco’s phosphate-buffered saline (DPBS) at a 1:4 (TB:DPBS) ratio. The dashed purple line marks three standard deviations (3*σ*) for the Gaussian fit of the histogram in panel **(D)**, which serves as a threshold point for counting dead cells in panels **(E)** and **(F)**. Scale bars: 100 *μ*m.

The final experiment was to use absorbance microscopy to count live and dead cells in the ‘Overgrown-5d’ and ‘DPBS-RT-5d’ samples (Fig 9), which mimic batches of low-viability cells that could be encountered during a cell manufacturing process. Following the cell counting strategy outlined in Section 2.6.5, the live-dead cutoff was determined as 1.64 fmol/cell (mean of the LT live-cell peak plus 3 standard deviations). Using this cutoff, ‘Overgrown-5d’ had 57% live and 43% dead cells, while ‘DPBS-RT-5d’ had 31% live and 69% dead cells (Fig 9E and 9F). The mean of the distribution attributed to living cells in Fig 9F is shifted to the right and is not centered around zero as in the LT and ‘Overgrown-5d’ cases shown in Fig 9D and 9E. The shift in TB uptake (Fig 9F) may indicate that the cells started degrading. The user must keep in mind that for these “naturally” dying cells, the dead cells may disintegrate and may not appear in the histograms as dead cell counts. Dead cells that disintegrate cannot be counted by TB staining.

## 4. Discussion

The results demonstrate a quantitative and traceable absorbance imaging method to measure intracellular TB uptake in mol/cell or mmol/L to improve the quality of live and dead cell classifications when assessing cell viability. There are several factors to consider when making these measurements.

### Unit (molarity vs. mol/cell)

The analysis of the results could be done in many different ways depending on user needs. The appropriate unit for analysis may be debated. Mol/cell and molarity were used herein (S5 Fig; Fig 6, Fig 7 and Fig 9.). Mol/cell could be misleading if a cell preparation has a large range of volumes. On the other hand, molarity requires cell volume calculations, which may introduce errors that can arise from microscope focusing variation, for example. When measured on two different microscopes, TB-stained heat-shock killed cells (LT) had a 21.9% difference in TB molarity but only a 3.8% difference in mol/cell (Fig 7). The difference between the TB molarity for the heat-shock killed cells on Microscope 1 versus Microscope 2 could be caused by the cell volume measurements. For the heat-shock experiments, the mean cell volume was 1.39±0.18 pL (n = 3 replicates, same sample preparation, data acquired on three different days) for Microscope 1 and 1.80±0.28 pL (n = 3 replicates) for Microscope 2, which is a difference of 22.8% (S6 Fig). Since molarity is equal to moles/volume, a 22.8% difference in volume nearly accounts for the 21.9% difference in TB molarity. The cause of the volume differences is unknown. There may have been actual differences in cell volume between the experiments possibly caused by differences in passage number or minor variations in culture conditions. They could also be caused by variability in the imaging. A small variation in the microscope focus or image properties can affect segmentation [24,25]. Since the semiaxes are cubed, a small effect on cell diameter can cause a large change in calculated volume. Here, the operator manually focused the images. The effect of focus variations on viability measurement error is recognized and improvements with reference beads are being developed [24,25].

The difference for heat-shock versus fixation-killed cells (Fig 7), however, is not due to differences in cell volume. The cell volumes (S6 Fig) for heat-shock Microscope 1 [1.39±0.18 pL (n = 3 replicates)] and fixation Microscope 1 [1.33±0.33 pL (n = 3 replicates)] differ by only ~4%. Instead, the killing method may affect how much TB is taken up by dead cells, where heat-shock-killed cells take in more TB.

### Threshold selection for live-dead classifications

TB mol/cell and molarity histograms enable the user to design a heuristic for making live and dead cell classifications that is fit for a given purpose and appropriate for the risk-benefit profile. Herein, the minimum, *C*_min_, between “Background & scattering subtracted” LT and DT histograms was selected as the threshold for making live and dead cell classifications (for fixation and heat shock treatments) (Fig 6). For example, if the application was a human cell therapy to treat articular cartilage and dead cells were thought to be harmful, then a heuristic that increases the likelihood of detecting dead cells could be employed. For example, setting the live-dead threshold at three SDs below the dead cell (DT) mean would capture 99.9% of dead cells (assuming a Gaussian distribution). Alternatively, for treating critically ill patients, maximizing live-cell delivery may be more of a concern than side effects from dead cells, in which case setting a threshold of three SDs above the live-cell mean (LT) would capture 99.9% of live cells. This approach was used to analyze ‘Overgrown-5d’ and ‘DPBS-RT-5d’ samples (Fig 9). Additionally, the intracellular levels of TB could be used to evaluate cell heath as a continuum, instead of making binary live/dead assignments, and could lead to more relevant TB-based assays [6].

### Measurement technique

Consistent measurement technique improves precision. Staining is dynamic and dead cells may take in more dye the longer they are incubated. Additionally, incubation of cells in DPBS and TB at room temperature may affect cell health. Mammalian cells prefer 37°C and their health may deteriorate at room temperature. Dyes may be toxic [15], so dead cell count may increase with longer TB staining times. These factors (staining time, temperature, exposure to TB and DPBS) may also affect cell volume, which will affect the molarity calculations. Finally, cells rapidly sediment, so gentle pipetting should be used to resuspend cells before imaging. In sum, staining and imaging should be performed in a rapid yet consistent manner.

Cell handling processes may be another source of variability when calculating live and dead cell populations (Fig 8). For instance, during an interlaboratory study using the MTT cell viability test (3-(4,5-dimethylthiazol-2-yl)-2,5-diphenyltetrazolium bromide), a mean intra-lab variability of 11% (variability within a single lab) was attributed to cell counting, pipetting, and seeding into culture plates [26]. The coefficient of variation for TB cell viability assessment of primary human bone marrow stromal cells over 60 replicate determinations of the same sample in the same lab was 4.4% using an automated imaging cell counter and 4.6% using a manual determination with a hemocytometer [27].

### Hardware and additional workload to implement absorbance imaging

The required hardware for absorbance imaging is a brightfield microscope. A monochromatic light source is needed as TB absorbance varies with wavelength (Fig 4A). The comparability of results between different microscope systems is improved when both systems use the same wavelength. If a bandpass filter is used, the user needs to be sure that it blocks a wide range of wavelengths to eliminate the possibility of far-red or far-blue light detection by the camera.

Four treatments (LD, DD, LT, DT) should be tested by the user to establish that the method is reliable for a given workflow. The four histograms for the three analytical methods shown in Fig 6 and S5 Fig could be generated and examined to determine how best to make the live and dead classifications. Once the method is established, routine use may only require LT and the test sample preparation. Also, another appropriate *I*_max_ for routine use might be a chamber slide filled with the medium containing TB (without cells), which would eliminate the need to subtract the background peak.

An open-source AbsorbanceQ app [19] is available for generating absorbance images from the brightfield images. Moreover, the generation of absorbance images may be incorporated into already existing automated imaging techniques used to assess cell viability.

### Other applications for absorbance imaging

Several light-absorbing proteins are expressed by humans including melanin, hemoglobin, and rhodopsin. Absorbance imaging may be useful for assessing the expression of these proteins during cell culture. Tissue-engineered retinal pigment epithelium, which expresses melanin, is being used to treat ocular diseases and pigmentation measurements may be useful as a non-invasive quality metric [20]. Synthetic blood may include biomanufactured red blood cells and their expression of hemoglobin by absorbance imaging could be a useful gauge of function. Many light-absorbing stains are used for histology. A well-known example is hematoxylin and eosin. Absorbance imaging could be used to generate traceable histologic micrographs where pixels were colored by moles of hematoxylin and moles of eosin, to improve comparability between different labs.

## 5. Conclusions

An absorbance microscopy approach is demonstrated for improving the traceability and comparability of cell viability measurements that use light-absorbing dyes. Comparability was demonstrated using ND filters with known OD values on four different microscopes, and the results from four different microscopes demonstrate a mean absolute deviation of 3% from the expected optical density values. Accuracy was assessed by determining the molar absorption coefficient (*ε*_610*nm*_) of TB solutions of known molarity using both absorbance imaging and a spectrophotometer. Results for *ε*_610*nm*_ for the two approaches differ by 2.1%. A data processing workflow for analyzing cells was developed that includes subtractions for background and scattering. Measurements of moles of TB taken up by heat-shock-killed Jurkat cells differed by 3.8% when measured on two different microscope systems. The method was tested on 50:50 mixtures of live:dead cells and the results demonstrated that 53% of cells were identified as dead (±6% SD). The method was also tested on two unknowns that were intended to mimic low viability cells that might be encountered during a cell manufacturing process: i) cells overgrown with no media change for five days in the cell culture incubator, and ii) cells incubated in DPBS at room temperature outside of the cell culture incubator for five days.

Absorbance microscopy is accessible to many researchers since the only hardware required is a brightfield microscope. An open-source AbsorbanceQ app is available for generating absorbance images. The value of absorbance imaging lies in having data with traceable units (mol/cell or molarity). Using the mol/cell or molarity histograms, the user can construct a mindful strategy for making live-dead classifications that accounts for the risk-benefit profile of the intended clinical indication. The traceability and comparability afforded by absorbance imaging are important when the goal is to biomanufacture numerous units of cell therapy products with consistent quality.

## Supporting information

S1 Fig Experimental setup. **(A)** Microscope equipped with λ = 610 nm bandpass filter (the black arrow points to the filter); **(B)** absorbance imaging of a 1 cm cuvette filled with trypan blue (TB) solution in Dulbecco’s phosphate-buffered saline (DPBS); **(C)** glass chamber slides containing a TB sample in position #1 for absorbance imaging.
(DOCX)

S2 Fig Optimizing microscope settings. For each microscope, the pixel intensity of DPBS blank reference (*I*_*max*_) was plotted against the shutter speed value. In this example, the shutter speed value of 9 ms was picked in the middle of the linear range (away from the saturation region to the right) after which the microscope settings were kept constant throughout the absorbance imaging process for each experiment.
(DOCX)

S3 Fig Calculations for determining “moles of trypan blue per pixel” in an absorbance image. (DOCX)

S4 Fig Fixation experiment. Brightfield intensity images and corresponding absorbance images of four Jurkat cell treatments. **(A,B)** LD: Live cells in Dulbecco’s phosphate-buffered saline (DPBS) solution; **(C,D)** LT: Live cells in trypan blue (TB) solution mixed with DPBS at a 1:4 (TB:DPBS) ratio; **(E,F)** DD: Dead cells in DPBS; **(G,H)** DT: Dead cells in TB solution mixed with DPBS at a 1:4 ratio (TB:DPBS). Dead cells were produced using fixation cell killing method. Arrowheads in panels C and D indicate a dead cell in the LT sample. The stars in panels D and H indicate the medium which contains TB solution mixed with DPBS at a 1:4 ratio (TB:DPBS) and absorbs light giving it a lighter shade of blue (higher absorbance value as compared to panels B and F). Scale bars: 100 *μ*m.
(DOCX)

S5 Fig Determination of intracellular trypan blue (TB) content (mmol/L) using absorbance images. Absorbance microscopy output data for **(A)** heat-shock and **(B)** fixation experiments illustrating the histograms of the intracellular TB molarity using three different image processing strategies: 1) ‘Raw moles,’ 2) ‘Background subtracted,’ and 3) ‘Background & scattering subtracted.’ The last column reveals the live and dead cell counts for the control ‘Live and dead cells in TB’ experiment, where an equal number of live and dead cells were mixed in TB+DPBS solution. The arrow in the last column denotes the calculated threshold using LT and DT samples using image processing strategy #3. The threshold is used to calculate live cells (all cells below the threshold) and dead cells (all cells above the threshold). Abbreviations: LD = live cells in Dulbecco’s phosphate-buffered saline (DPBS) solution, LT = live cells in trypan blue (TB) solution mixed with DPBS at a 1:4 (TB:DPBS) ratio, DD = dead cells in DPBS, and DT = dead cells in TB solution mixed with DPBS at a 1:4 ratio (TB:DPBS). The purple solid lines represent the Gaussian fit which yields the mean (*μ*) and the standard deviation (*σ*) of intracellular TB concentration. The histograms in panels **(A)** and **(B)** show that the intracellular uptake of TB is different depending on the cell killing method.
(DOCX)

S6 Fig Cell volumes (DT treatment) determined by image analysis of absorbance images. Open symbols indicate triplicate experiments (same sample preparation, data acquired on three different days) of heat-shock treatment using Microscope 1 (black circles), heat-shock treatment using Microscope 2 (blue triangles), and fixation treatment using Microscope 1 (red squares). Microscope 1: Biotek Lionheart FX, Microscope 2: Nikon Ti2. The filled symbols represent the mean of three replicates. The error bars represent the standard deviation. *P*-values from t-tests are shown below brackets.
(DOCX)
