# Supplementary material for: Quantitative, traceable determination of cell viability using absorbance microscopy
Source: PLoS One. 2022 Jan 19;17(1):e0262119. doi: 10.1371/journal.pone.0262119 (PMC8769294; doi:10.1371/journal.pone.0262119)
Supplement: S1 Fig — (A) Microscope equipped with λ = 610 nm bandpass filter (the black arrow points to the filter); (B) absorbance imaging of a 1 cm cuvette filled with trypan blue (TB) solution in Dulbecco’s phosphate-buffered saline (DPBS); (C) glass chamber slides containing a TB sample in position #1 for absorbance imaging. (DOCX) [file pone.0262119.s001.docx]

**
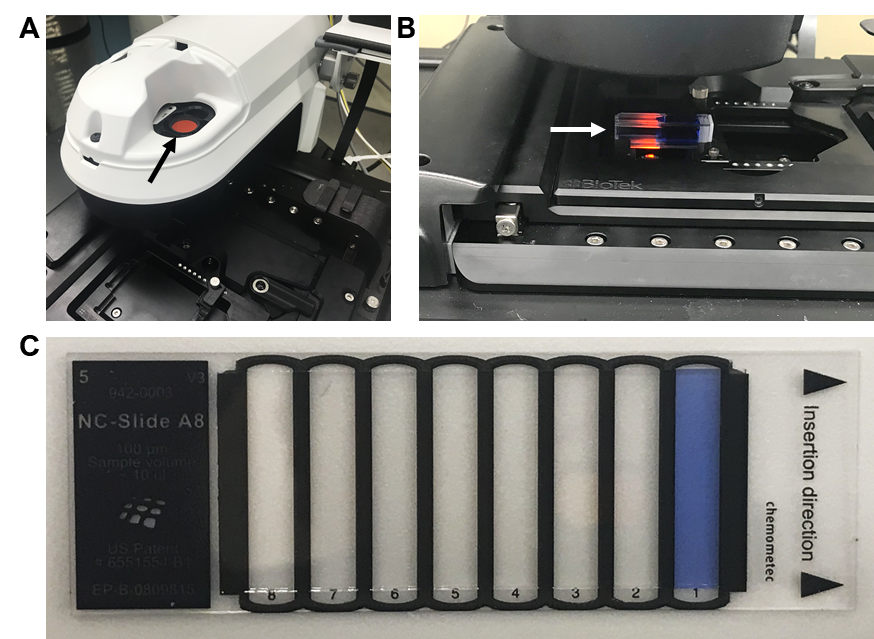
**

**Fig S1.** **Experimental setup. (A)** Microscope equipped with λ = 610 nm bandpass filter (the black arrow points to the filter); **(B)** absorbance imaging of a 1 cm cuvette filled with trypan blue (TB) solution in Dulbecco's phosphate-buffered saline (DPBS); **(C)** glass chamber slides containing a TB sample in position #1 for absorbance imaging.
