# Supplementary material for: Quantitative, traceable determination of cell viability using absorbance microscopy
Source: PLoS One. 2022 Jan 19;17(1):e0262119. doi: 10.1371/journal.pone.0262119 (PMC8769294; doi:10.1371/journal.pone.0262119)
Supplement: S2 Fig — For each microscope, the pixel intensity of DPBS blank reference (Imax) was plotted against the shutter speed value. In this example, the shutter speed value of 9 ms was picked in the middle of the linear range (away from the saturation region to the right) after which the microscope settings were kept constant throughout the absorbance imaging process for each experiment. (DOCX) [file pone.0262119.s002.docx]

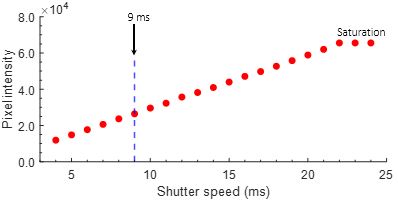


**Fig S2.** **Optimizing microscope settings.** For each microscope, the pixel intensity of DPBS blank reference (*I_max_*) was plotted against the shutter speed value. In this example, the shutter speed value of 9 ms was picked in the middle of the linear range (away from the saturation region to the right) after which the microscope settings were kept constant throughout the absorbance imaging process for each experiment.
