# Supplementary material for: Quantitative, traceable determination of cell viability using absorbance microscopy
Source: PLoS One. 2022 Jan 19;17(1):e0262119. doi: 10.1371/journal.pone.0262119 (PMC8769294; doi:10.1371/journal.pone.0262119)
Supplement: S3 Fig — (DOCX) [file pone.0262119.s003.docx]

**Fig S3.** **Calculations for determining “moles of trypan blue per pixel” in an absorbance image.**

- **Variables**
- *c* = Concentration = $\frac{A}{\varepsilon l}$ (Beer-Lambert Law)
- *V* = Volume = $xyz$
- $x$ = width = this depends on the pixel width in the image; for Microscope #1 the pixel width was 0.000000658 m which is the value used in the calculation below
- *y* = length = this depends on the pixel width in the image; for Microscope #1 the pixel width was 0.000000658 m which is the value used in the calculation below
- *z* = depth = 0.01 m (pathlength)
- $A$ = Absorbance = This is the value that is measured for each pixel in an absorbance image using Eq.1.
- $\varepsilon$ = Molar absorption coefficient = 2609 m^2^/mol (measured at 610 nm wavelength)
- *Notes:*
  - In Section 3.2 and Fig. 4, $\varepsilon$ was determined to be 2609 m^2^/mol.
  - $\varepsilon$ can be expressed as (m^2^/mol) or as (M^−1^⋅cm^−1^ or L⋅mol^−1^⋅cm^−1^), the latter of which are equal to 0.1 m^2^/mol.
- $l$ = Pathlength = 0.01 m

*Note:* A 0.01 m value is used for the pathlength so that the moles in the entire pathlength are determined. The cell volume, which is determined later in the algorithm, is used to calculate intracellular molarity.

- **Equations**
  - *Equation for calculating moles:*

Moles = [*c* × *V*] = [(mol/L) × *V*] = $\frac{A}{\varepsilon l}\left( xyz \right)$

- - *Plug in Values:*

Moles = $\frac{A}{2609\frac{m^{2}}{\mathrm{mol}} \times0.01 m} \times0. 000000658 m \times0. 000000658 m \times0.01 m$

- - *Final conversion equation from absorbance values to moles:*

Moles = $A \times1.660 \times{10}^{-16} \mathrm{moles}$

(for image with pixels dimensions 0.000000658 m $\times$0.000000658 m)
