# Supplementary material for: Quantitative, traceable determination of cell viability using absorbance microscopy
Source: PLoS One. 2022 Jan 19;17(1):e0262119. doi: 10.1371/journal.pone.0262119 (PMC8769294; doi:10.1371/journal.pone.0262119)
Supplement: S4 Fig — Brightfield intensity images and corresponding absorbance images of four Jurkat cell treatments. (A,B) LD: Live cells in Dulbecco’s phosphate-buffered saline (DPBS) solution; (C,D) LT: Live cells in trypan blue (TB) solution mixed with DPBS at a 1:4 (TB:DPBS) ratio; (E,F) DD: Dead cells in DPBS; (G,H) DT: Dead cells in TB solution mixed with DPBS at a 1:4 ratio (TB:DPBS). Dead cells were produced using fixation cell killing method. Arrowheads in panels C and D indicate a dead cell in the LT sample. The stars in panels D and H indicate the medium which contains TB solution mixed with DPBS at a 1:4 ratio (TB:DPBS) and absorbs light giving it a lighter shade of blue (higher absorbance value as compared to panels B and F). Scale bars: 100 μm. (DOCX) [file pone.0262119.s004.docx]

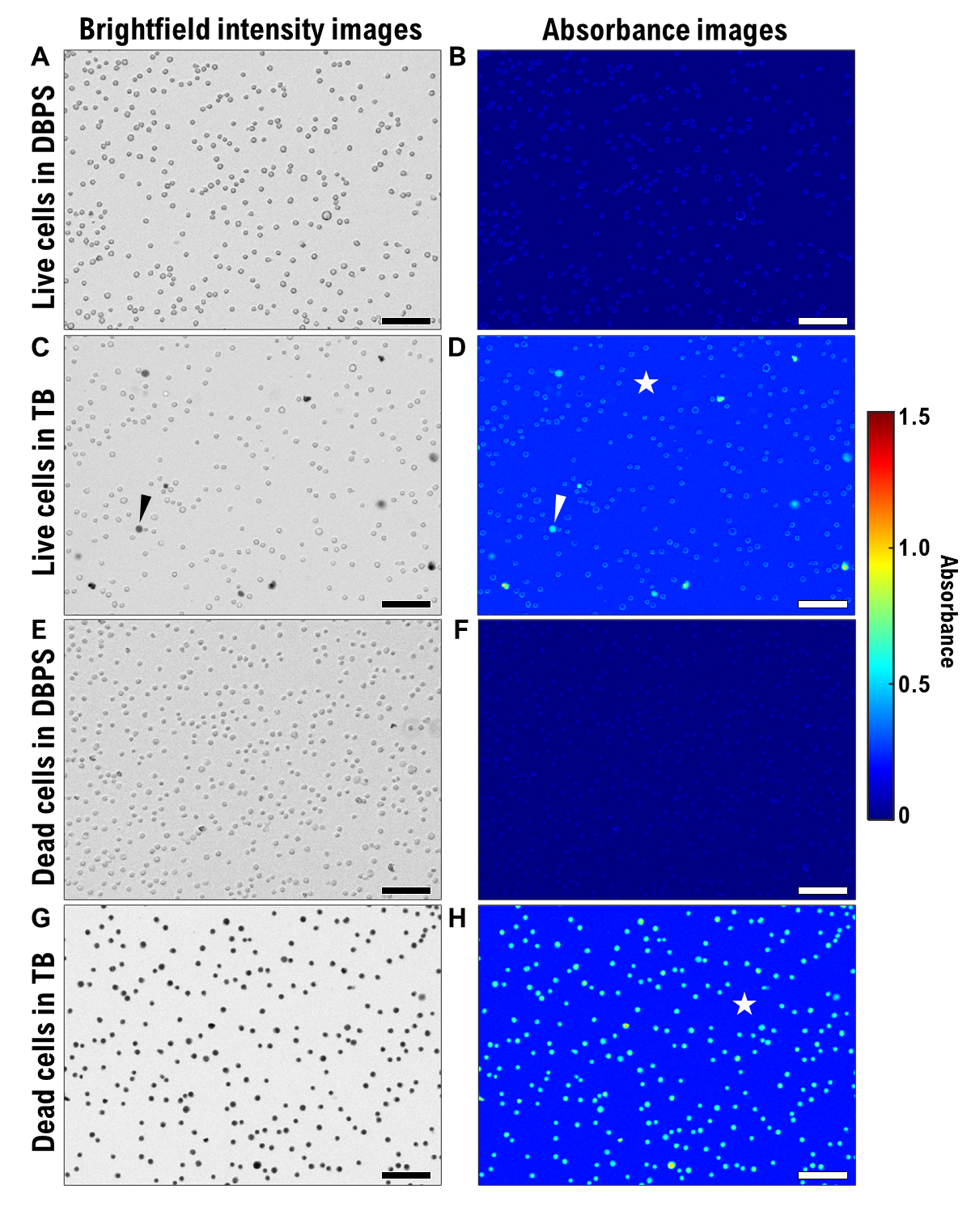


**Fig S4.** **Fixation experiment.** Brightfield intensity images and corresponding absorbance images of four Jurkat cell treatments. **(A,B)** LD: live cells in Dulbecco's phosphate-buffered saline (DPBS) solution; **(C,D)** LT: live cells in trypan blue (TB) solution mixed with DPBS at a 1:4 (TB:DPBS) ratio; **(E,F)** DD: dead cells in DPBS; **(G,H)** DT: dead cells in TB solution mixed with DPBS at a 1:4 ratio (TB:DPBS). Dead cells were produced using fixation cell killing method. Arrowheads in panels C and D indicate a dead cell in the LT sample. The stars in panels D and H indicate the medium which contains TB solution mixed with DPBS at a 1:4 ratio (TB:DPBS) and absorbs light giving it a lighter shade of blue (higher absorbance value as compared to panels B and F). Scale bars: 100 *µ*m.
