# Supplementary material for: Quantitative, traceable determination of cell viability using absorbance microscopy
Source: PLoS One. 2022 Jan 19;17(1):e0262119. doi: 10.1371/journal.pone.0262119 (PMC8769294; doi:10.1371/journal.pone.0262119)
Supplement: S5 Fig — Absorbance microscopy output data for (A) heat-shock and (B) fixation experiments illustrating the histograms of the intracellular TB molarity using three different image processing strategies: 1) ‘Raw moles,’ 2) ‘Background subtracted,’ and 3) ‘Background & scattering subtracted.’ The last column reveals the live and dead cell counts for the control ‘Live and dead cells in TB’ experiment, where an equal number of live and dead cells were mixed in TB+DPBS solution. The arrow in the last column denotes the calculated threshold using LT and DT samples using image processing strategy #3. The threshold is used to calculate live cells (all cells below the threshold) and dead cells (all cells above the threshold). Abbreviations: LD = live cells in Dulbecco’s phosphate-buffered saline (DPBS) solution, LT = live cells in trypan blue (TB) solution mixed with DPBS at a 1:4 (TB:DPBS) ratio, DD = dead cells in DPBS, and DT = dead cells in TB solution mixed with DPBS at a 1:4 ratio (TB:DPBS). The purple solid lines represent the Gaussian fit which yields the mean (μ) and the standard deviation (σ) of intracellular TB concentration. The histograms in panels (A) and (B) show that the intracellular uptake of TB is different depending on the cell killing method. (DOCX) [file pone.0262119.s005.docx]

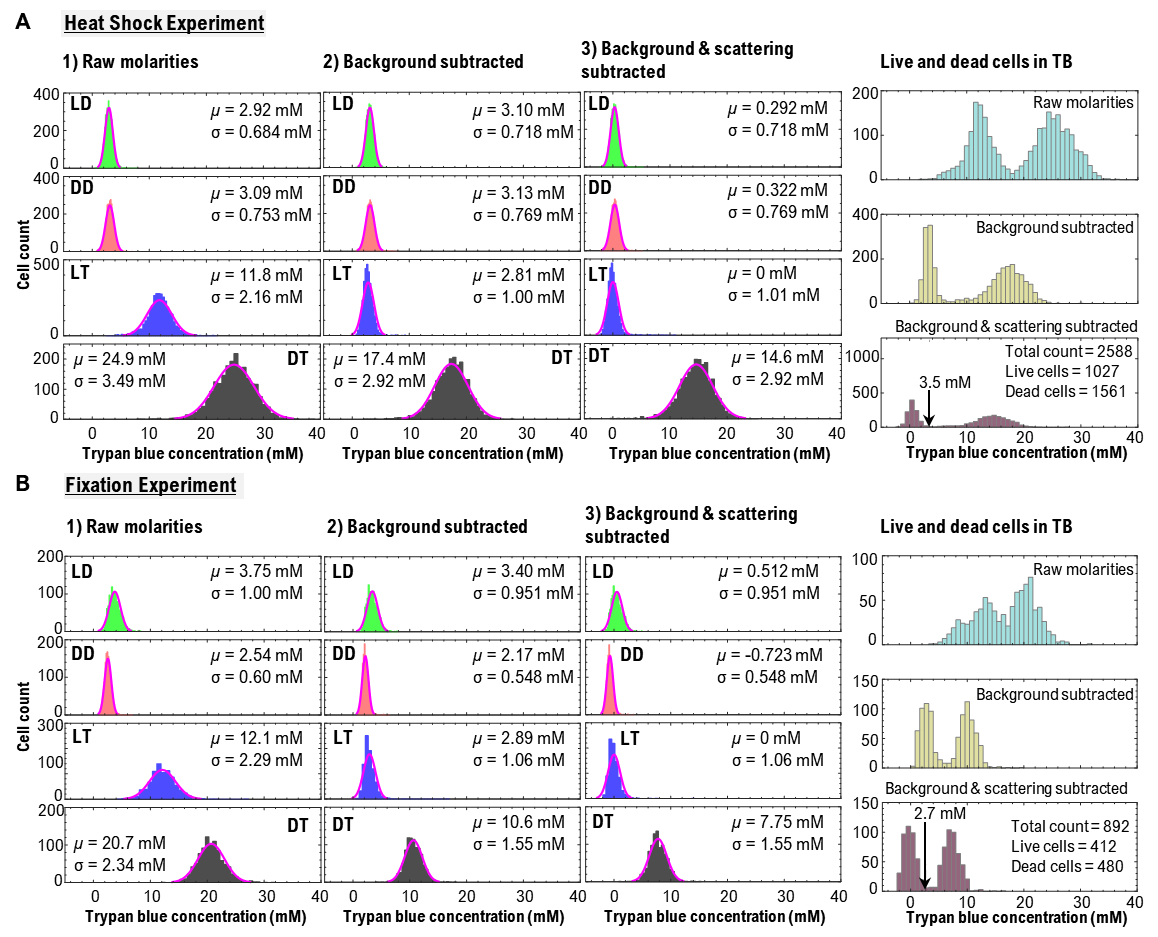


**Fig S5.** **Determination of intracellular trypan blue (TB) content (mmol/L) using absorbance images.** Absorbance microscopy output data for **(A)** heat-shock and **(B)** fixation experiments illustrating the histograms of the intracellular TB molarity using three different image processing strategies: 1) ‘Raw moles,’ 2) ‘Background subtracted,’ and 3) ‘Background & scattering subtracted.’ The last column reveals the live and dead cell counts for the control ‘Live and dead cells in TB’ experiment, where an equal number of live and dead cells were mixed in TB+DPBS solution. The arrow in the last column denotes the calculated threshold using LT and DT samples using image processing strategy #3. The threshold is used to calculate live cells (all cells below the threshold) and dead cells (all cells above the threshold). Abbreviations: LD = live cells in Dulbecco's phosphate-buffered saline (DPBS) solution, LT = live cells in trypan blue (TB) solution mixed with DPBS at a 1:4 (TB:DPBS) ratio, DD = dead cells in DPBS, and DT = dead cells in TB solution mixed with DPBS at a 1:4 ratio (TB:DPBS). The purple solid lines represent the Gaussian fit which yields the mean ($\mu$) and the standard deviation ($\sigma$) of intracellular TB concentration. The histograms in panels **(A)** and **(B)** show that the intracellular uptake of TB is different depending on the cell killing method.
