# Supplementary material for: Quantitative, traceable determination of cell viability using absorbance microscopy
Source: PLoS One. 2022 Jan 19;17(1):e0262119. doi: 10.1371/journal.pone.0262119 (PMC8769294; doi:10.1371/journal.pone.0262119)
Supplement: S6 Fig — Open symbols indicate triplicate experiments (same sample preparation, data acquired on three different days) of heat-shock treatment using Microscope 1 (black circles), heat-shock treatment using Microscope 2 (blue triangles), and fixation treatment using Microscope 1 (red squares). Microscope 1: Biotek Lionheart FX, Microscope 2: Nikon Ti2. The filled symbols represent the mean of three replicates. The error bars represent the standard deviation. P-values from t-tests are shown below brackets. (DOCX) [file pone.0262119.s006.docx]

**
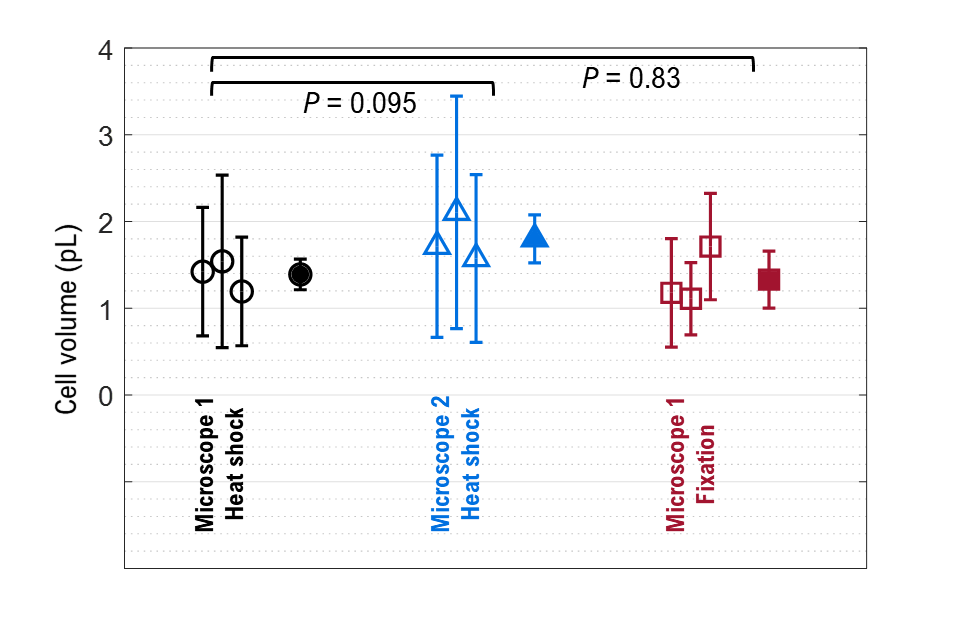
**

**Fig S6.** **Cell volumes (DT treatment) determined by image analysis of absorbance images.** Open symbols indicate triplicate experiments (same sample preparation, data acquired on three different days) of heat-shock treatment using Microscope 1 (black circles), heat-shock treatment using Microscope 2 (blue triangles), and fixation treatment using Microscope 1 (red squares). Microscope 1: Biotek Lionheart FX, Microscope 2: Nikon Ti2. The filled symbols represent the mean of three replicates. The error bars represent the standard deviation. *P*-values from t-tests are shown below brackets.
